# Conserved Residue Asn-145 in the C-Terminal Heptad Repeat Region of HIV-1 gp41 is Critical for Viral Fusion and Regulates the Antiviral Activity of Fusion Inhibitors

**DOI:** 10.3390/v11070609

**Published:** 2019-07-03

**Authors:** Xiuzhu Geng, Zixuan Liu, Danwei Yu, Bo Qin, Yuanmei Zhu, Sheng Cui, Huihui Chong, Yuxian He

**Affiliations:** 1 NHC Key Laboratory of Systems Biology of Pathogens, Institute of Pathogen Biology, Chinese Academy of Medical Sciences and Peking Union Medical College, Beijing 100730, China; 2 Center for AIDS Research, Chinese Academy of Medical Sciences and Peking Union Medical College, Beijing 100730, China

**Keywords:** HIV-1, gp41, membrane fusion, six-helical bundle, fusion inhibitor

## Abstract

Entry of HIV-1 into target cells is mediated by its envelope (Env) glycoprotein composed of the receptor binding subunit gp120 and the fusion protein gp41. Refolding of the gp41 N- and C-terminal heptad repeats (NHR and CHR) into a six-helix bundle (6-HB) conformation drives the viral and cellular membranes in close apposition and generates huge amounts of energy to overcome the kinetic barrier leading to membrane fusion. In this study, we focused on characterizing the structural and functional properties of a single Asn-145 residue, which locates at the middle CHR site of gp41 and is extremely conserved among all the HIV-1, HIV-2, and simian immunodeficiency virus (SIV) isolates. By mutational analysis, we found that Asn-145 plays critical roles for Env-mediated cell-cell fusion and HIV-1 entry. As determined by circular dichroism (CD) spectroscopy and isothermal titration calorimetry (ITC), the substitution of Asn-145 with alanine (N145A) severely impaired the interactions between the NHR and CHR helices. Asn-145 was also verified to be important for the antiviral activity of CHR-derived peptide fusion inhibitors and served as a turn-point for the inhibitory potency. Intriguingly, Asn-145 could regulate the functionality of the M-T hook structure at the N-terminus of the inhibitors and displayed comparable activities with the C-terminal IDL anchor. Crystallographic studies further demonstrated the importance of Asn-145-mediated interhelical and intrahelical interactions in the 6-HB structure. Combined, the present results have provided valuable information for the structure-function relationship of HIV-1 gp41 and the structure-activity relationship of gp41-dependent fusion inhibitors.

## 1. Introduction

**Figure 1:**
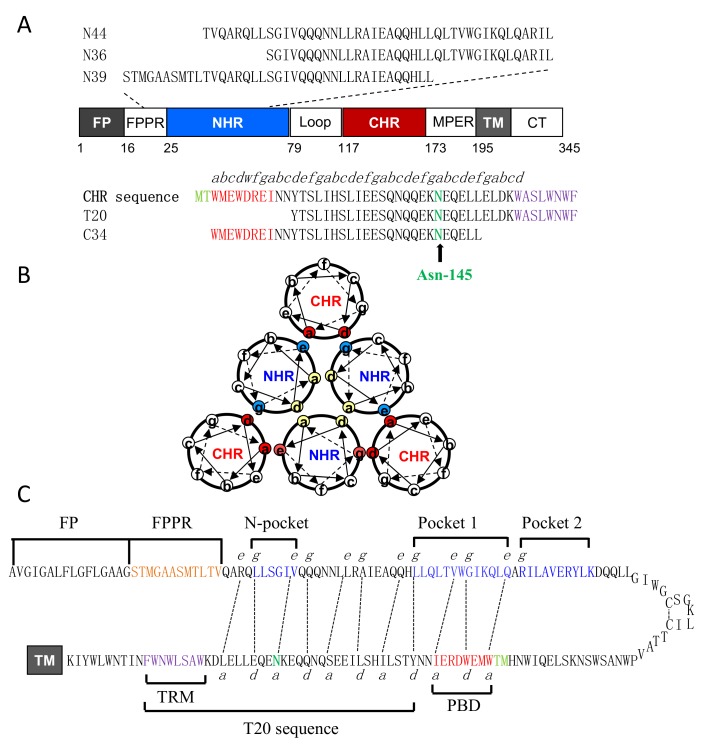
Schematic diagram of HIV-1 gp41 and 6-HB structure. (**A**) Functional domains of HIV-1 gp41 and its NHR- and CHR-derived peptides. The gp41 numbering of HIV-1_HXB2_ is used. FP, fusion peptide; FPPR, fusion peptide proximal region; NHR, N-terminal heptad repeat; CHR, C-terminal heptad repeat; TM, transmembrane domain; CT, cytoplasmic tail. The italic “*abcdefg*” on the CHR sequence corresponds to the positions of the amino acids per heptad repeat. The M-T hook residues, pocket-binding domain (PBD), Asn-145 residue, and tryptophan-rich motif (TRM) are respectively marked in aqua, red, green, and purple. (**B**) The helical wheel model illustrating the interactions between the NHR and CHR helices of gp41. The inner NHR homotrimer is packed through the interactions of residues at the “*a*” and “*d*” positions (marked in yellow), whereas its residues at the “*e*” and “*g*” positions (in aquamarine) on the outside of the coiled-coil contact with the residues at the “*a*” and “*d*” positions (in red) of the CHR helices. (**C**) The hairpin model illustrating the interacting residues between the NHR and CHR sequences. The dashed black lines indicate the interactions between the residues at the “*e*” and “*g*” positions in the NHR and the “*a*” and “*d*” positions in the CHR, respectively. The sequences of FP, FPPR, N-pocket, and C-pockets (pocket-1 and pocket-2) in the NHR and PBD and T20 in the CHR are respectively marked for clarity.

Two envelope (Env) glycoproteins of human immunodeficiency virus type 1 (HIV-1) coordinately catalyze membrane fusion between the virus and target cells [1,2]. Accompanying with a cascade of conformational changes, the surface subunit gp120 is responsible for binding with the cell receptor CD4 and a coreceptor (CCR5 or CXCR4), whereas the transmembrane subunit gp41 is involved in fusion reaction. As illustrated in Figure 1, the sequence structure of gp41 can be divided into multiple functional domains: fusion peptide (FP), FP proximal polar region (FPPR), N-terminal heptad repeat (NHR), loop region, C-terminal heptad repeat (CHR), membrane proximal external region (MPER), transmembrane domain (TM), and long cytoplasmic tail (CT). Upon receptor binding, the gp41 transiently undergoes an extended, membrane-bridging prehairpin state with its FP being inserted into the cell membrane; then, three CHRs fold on the trimeric coiled coil of NHR in a antiparallel manner, assembling a stable six-helix bundle (6-HB) that drives the viral and cell membranes in close apposition for fusion [3,4]. Peptides derived from either NHR or CHR of gp41 can bind to the prehairpin state and competitively block viral 6-HB formation, thereby inhibiting Env-mediated cell fusion and HIV-1 entry in a dominant negative fashion [2,5]. As a milestone, the CHR-derived peptide T20 (enfuvirtide) was clinically approved in 2003 as the first HIV-1 fusion/entry inhibitor, which is used in combination therapy of HIV-1 infection [6,7]. Considering that the 6-HB structure represents a typical structural feature of class I fusion glycoproteins employed by many enveloped viruses, the success of T20 spurred tremendous efforts to exploit the mechanisms of viral membrane fusion and inhibition [1,8].

A substantial body of evidence supports that the interactions between the NHR and CHR helices essentially determine the functionality of viral 6-HB structure [3,4,9,10,11,12,13]. Structurally, the coiled-coil domains HIV-1 gp41 share a characteristic heptad repeat sequence, usually denoted as (*abcdefg*)*n*, with hydrophobic residues at the first (*a*) and fourth (*d*) positions. In a helical wheel model (Figure 1), the inner NHR homotrimer is packed against each other through the interactions of residues at the “*a*” and “*d*” sites, whereas its residues at the “*e*” and “*g*” sites lie on the outside of the central coiled-coil to make extensive contacts with the residues at the “*a*”, “*d*”, and “*e*” sites of the CHR helices. Prominently, the residues at the “*b*”, “*c*”, and “*f*” sites on both the NHR and CHR helices are located away from the interaction center. In terms of the sequence, the gp41 CHR can be further divided into an N-terminal pocket-binding domain (PBD), a middle NHR-binding domain (NBD), and a C-terminal tryptophan-rich motif (TRM). Crystal structures of the 6-HBs reveal that three hydrophobic residues from the PBD (Ile-117, Trp-120, and Trp-124) penetrate a deep pocket on the C-terminal portion of the NHR trimer, which critically stabilizes the NHR-CHR interactions [3,4,9,14,15].

Previously, we endeavored to characterize the structural properties of gp41 and achieved significant findings. First, a highly conserved salt-bridge was identified between the negatively charged Asp-127 in the PBD of CHR and the positively charged Lys-63 in the pocket of NHR [16,17]; second, the residues Met-125 and Thr-126 preceding the PBD was found to adopt a unique hook-like structure (designated M-T hook) [18,19,20]; third, a subpocket (designated pocket-2) was defined by seven residues on one NHR helix and four residues on another NHR helix [21]; Very recently, we found the critical interactions between the TRM of CHR and the FPPR of NHR [22]. All these structural features were verified to be essential in gp41-mediated viral fusion, providing important information for mechanism of HIV-1 cell fusion and for developing novel HIV-1 inhibitors. In this study, we focused on the polar residue Asn-145 on the basis of our following observations. Asn-145 is an extremely conserved residue among all the HIV-1, HIV-2, and simian immunodeficiency virus (SIV) isolates (www.hiv.lanl.gov), but its functional properties are not clear. In the native Env structure (prefusion form), Asn-145 locates the α9 helix formed by the CHR sequence, but its potential interaction and functionality cannot be finely defined [23,24,25]. In the helical wheel and hairpin models (Figure 1B,C), Asn-145 locates at the “*a*” position in the CHR helix and interacts with Val-38 at the “*e*” position of the NHR helix, which is a key component of the “GIV” motif that critically determines T20 resistance. Recently, Asn-145 was intentionally substituted by a C-terminal IDL anchor to generate fusion inhibitors that can target a newly-defined shallow pocket at the N-terminal site of the NHR trimer [26,27,28]. We have comprehensively characterized Asn-145 from multiple angles and demonstrated its important roles for Env-mediated HIV-1 entry and fusion inhibitors.

## 2. Materials and Methods

### 2.1. Plasmids and Cell Lines

The following reagents were obtained through the AIDS Reagent Program, Division of AIDS, NIAID, NIH: the plasmid encoding the Env of HIV-1 SVPB13 (AC10.29) from David Montefiori and Feng Gao; the plasmid encoding the Env of HIV-1 SVPC13 (ZM109F.PB4) from Cynthia A. Derdeyn and Eric Hunter; TZM-bl indicator cells stably expressing CD4 and CCR5 along with endogenously expressed CXCR4 from John C. Kappes and Xiaoyun Wu. The plasmid expressing DSP_1-7_ and 293FT cells stably expressing CXCR4/CCR5 and DSP_8–11_ were provided by Zene Matsuda at the Institute of Medical Science of the University of Tokyo (Tokyo, Japan). HEK293T cells were purchased from the American type culture collection (ATCC) (Rockville, MD, USA). The cell lines were cultured in complete growth medium that consisted of Dulbecco’s minimal essential medium (DMEM) supplemented with 10% fetal bovine Serum, 100 U/mL of Penicillin-Streptomycin, 2 mM L-Glutamine, 1mM Sodium Pyruvate, and 1 x MEM nonessential Amino Acids (Gibco/Invitrogen, Grand Island, New York, USA) and were maintained at 37 ℃ in 5% CO_2_.

### 2.2. Peptide Synthesis

Peptides were synthesized on rink amide 4-methylbenzhydrylamine (MBHA) resin by using a standard solid-phase 9-fluorenylmethoxy carbonyl (FMOC) method as described previously [29]. All the peptides were N-terminally acetylated and C-terminally amidated. They were purified by reverse-phase high-performance liquid chromatography (HPLC) to a purity of >95% and characterized with mass spectrometry for correct amino acid composition. Concentrations of the peptides were quantitated by UV absorbance and a theoretically calculated molar-extinction coefficient based on tryptophan and tyrosine residues.

### 2.3. Site-Directed Mutagenesis

HIV-1 Env mutants were generated by site-directed mutagenesis, as described previously [30]. In brief, two primers were designed with the desired mutation and located the same starting and ending positions on opposite strands of the template plasmid. DNA synthesis was conducted by PCR in a 20-μL reaction volume using 20 pM upper and lower primers, 100 ng of template plasmid, and 2 U of the high-fidelity polymerase PrimeStar (TaKaRa, Dalian, China). PCR amplification was carried out for one cycle of denaturation at 98 ℃ for 5 min, followed by 25 cycles of 98 ℃ for 15 s and 68 ℃ for 9 min, with a final extension at 72 ℃ for 10 min. After the amplicons were treated with DpnI for 1 h at 37 ℃ digestion-resistant plasmids were recovered by transforming *Escherichia coli* strain DH5α with antibiotic resistance. The successful mutations were confirmed by DNA sequencing.

### 2.4. Single-Cycle Infection Assay

HIV-1 entry was determined by a single-cycle infection assay as described previously [30]. Briefly, Env-based pseudoviruses were generated by cotransfecting HEK293T cells with an Env-expressing plasmid and a backbone plasmid pSG3^ΔENV^ that has an Env-defective, luciferase-expressing HIV-1 genome. Virus supernatants were harvested 48 h after transfection, and 50% tissue culture infectious doses (TCID_50_) were determined in TZM-bl cells. To evaluate the infectivity of pseudoviruses, the same amount of viral particles was normalized by p24 antigen. To measure the antiviral activity of fusion inhibitors, peptides were prepared in graded concentrations, mixed with 100 TCID_50_ of viruses, and then incubated 1 h at room temperature. The mixture was added to TZM-bl cells (10^4^/well) and then incubated 48 h at 37 ℃. Luciferase activity was measured using luciferase assay reagents and a luminescence counter (Promega, Madison, WI, USA). Percent inhibition of the pseudovirus by an inhibitor and 50% inhibitory concentration (IC_50_) were calculated using GraphPad Prism software (GraphPad Software Inc., San Diego, CA, USA).

### 2.5. Cell-Cell Fusion Assay

A dual split-protein (DSP)-based cell-cell fusion assay was used to measure Env-mediated cell-cell fusion activity as described previously [30]. Briefly, a total of 1.5 × 10^4^ HEK293T cells (effector cells) were seeded on a 96-well plate. On the following day, HEK293T cells were transfected with a mixture of an Env-expressing plasmid and a DSP_1-7_ plasmid. At 24h post transfection, 3 × 10^4^ 293FT stably expressing CXCR4/CCR5 and DSP_8-11_ (target cells) were resuspended in prewarmed culture medium containing EnduRen live-cell substrate (Promega) at a final concentration of 17 ng/μL and then transferred to each well of the effector cells at equal volumes. After the cell mixture was spun down, the luciferase activity was measured as described above.

As described previously [31], the inhibitory activity of peptides on HIV-1_HXB2_ Env-mediated cell-cell fusion was measured using a reporter gene assay based on the activation of an HIV-1 long terminal repeat (LTR)-driven luciferase cassette in target cells by HIV-1 tat from HL2/3 cells (effector). Briefly, TZM-bl cells (target) were plated in 96-well plates (1 × 10^4^/well) and incubated at 37 ℃ overnight. Then, 3 × 10^4^/well of HL2/3 cells were cocultured with target cells for 6 h at 37 ℃ in the presence or absence of a peptide inhibitor at graded concentrations, followed by the measurement of the luciferase activity.

### 2.6. Western Blotting Assay

The expression and processing profile of HIV-1 Env glycoproteins were examined by Western blotting as described previously [32]. Briefly, HEK293T cells were transfected with an Env-encoding plasmid. Cell lysates were centrifuged at 20,000× *g* at 4 ℃ for 15 min to remove insoluble materials. Equal amounts of total proteins were separated by SDS-PAGE and then transferred to a nitrocellulose membrane. After blocking with 5% nonfat dry milk solution in Tris-buffered saline (TBS, pH 7.4) at room temperature for 1 h, the membrane was incubated with a rabbit anti-gp120 polyclonal antibody (SinoBiological, Beijing, China) or the human anti-gp41 monoclonal antibody 10E8 overnight at 4 ℃. After washing three times with TBS-Tween 20, the membrane was incubated with IRDye 680LT goat-anti-rabbit IgG or IRDye 800CW goat-anti-human IgG for 2 h at room temperature. Images were obtained by scanning the membrane using the Odyssey infrared imaging system (LI-COR Biosciences, Lincoln, NE, USA).

### 2.7. Circular Dichroism (CD) Spectroscopy

Secondary structure and thermostability of the peptide complexes were determined by circular dichroism (CD) spectroscopy as described previously [30]. Briefly, 10 µM of a CHR-derived peptide was incubated with an equal molar concentration of an NHR-derived target mimic peptide at 37 ℃ for 30 min in phosphate-buffered saline (PBS, pH 7.2). CD spectra were then obtained on a Jasco spectropolarimeter (model J-815) using a 1-nm bandwidth with a 1-nm step resolution from 195 to 260 nm at 20 ℃. The α-helical content was calculated from the CD signal by dividing the mean residue ellipticity [*θ*] at 222 nm by the value expected for 100% helix formation (−33,000 degrees cm^2^·dmol^−1^). Thermal denaturation was performed by monitoring the ellipticity change at 222 nm from 20 to 98 ℃ at a rate of 2 ℃/min using a temperature controller and melting temperature (*T*_m_) was defined as the midpoint of the thermal unfolding transition.

### 2.8. Isothermal Titration Calorimetry (ITC) Experiment

The interaction affinity between the NHR and CHR peptides was determined by isothermal titration calorimetry **(**ITC) as described previously [18]. Thermodynamic parameters were acquired on an ITC-200 Microcalorimeter instrument (MicroCal, Northampton, Massachusetts, USA). In brief, 1 mM of an NHR-derived target mimic peptide (N39 or N36) was dissolved in double distilled H_2_O and injected into a chamber containing 80 μM of a CHR-derived inhibitor peptide (T20 and its N145A mutant, C34 and its N145A mutant, SC29EK, or SC28EK). The time between injections was 480 s and the stirring speed was 400 rpm. The experiments were conducted at 25 ℃. Data acquisition and analysis were carried out using MicroCal Origin software (version 7.0).

### 2.9. Crystallization and Structure Determination

The complex of SC29EK and N44 was prepared by dissolving an equal amount (1:1 molar ratio) of the peptides in a denaturing buffer (100 mM NaH_2_PO_4_, 10 mM Tris-HCl, pH 8.0, 8 M urea). The mixture was dialyzed against the buffer containing 50 mM Tris-HCl with a pH 8.0 and 100 mM NaCl at 4 ℃ overnight, and then applied onto Superdex-75 gel filtration column (GE Healthcare, Piscataway, NJ, USA) to collect the peak corresponding to the 6-HB size. The SC29EK/N44 complex was then concentrated to 20 mg/mL and mixed with an equal volume of reservoir solution containing 0.2 M zinc acetate hydrate (pH 5.6) and 18% (*w/v*) PEG 3350. After two days, single crystals were soaked in a reservoir solution containing 15% glycerol, followed by flash freezing in liquid nitrogen for 30–60 s. The diffraction data sets of SC29EK/N44 complex crystals were collected at beamline BL-17U1 at the Shanghai Synchrotron Radiation Facility, China. X-ray diffraction data were integrated and scaled using the XDS program [33]. The phasing problem was solved by the molecular replacement method using CCP4i with a crystal structure of HIV-1 gp41 6-HB (Protein Data Bank [PDB] accession number 5H0N) as a search model. The coordinates were deposited in the PDB under accession number 6J5E. All structural models were generated with PyMol (https://pymol.org/). The statistics of data collection and structure refinement are given in Table 2.

## 3. Results

### 3.1. Asn-145 is Critical for HIV-1 Env-Mediated Cell Fusion

**Figure 2:**
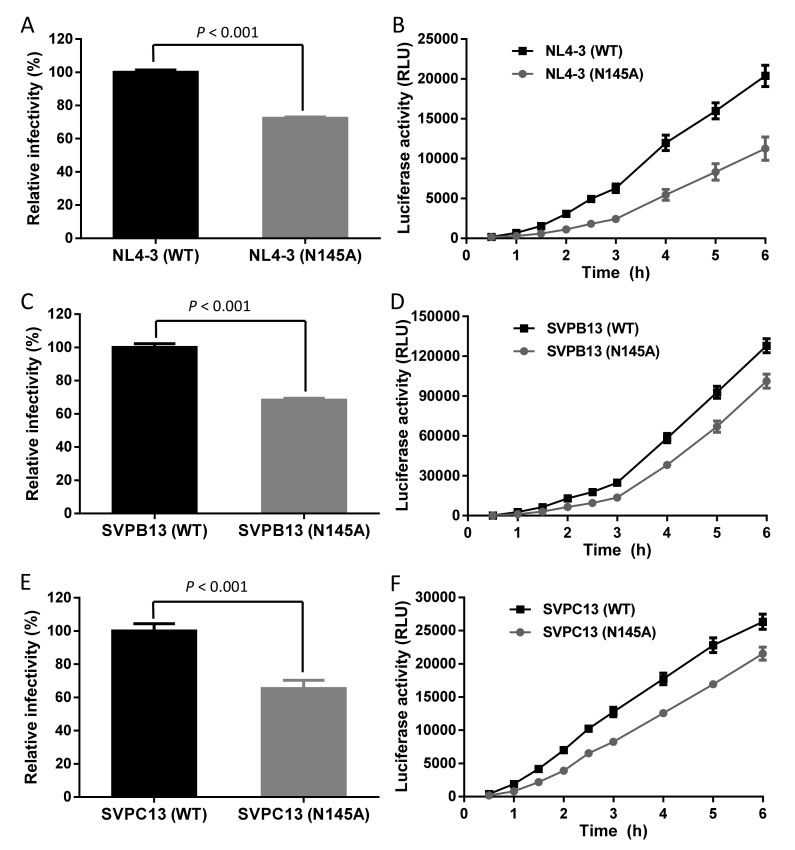
Effects of the N145A mutation on the functionality of HIV-1 Env. The N145A mutation severely impairs the HIV-1_NL4-3_ pseudovirus entry (**A**) and Env-mediated cell-cell fusion (**B**), HIV-1_SVPB13_ pseudovirus entry (**C**) and Env-mediated cell-cell fusion (**D**), and HIV-1_SVPC13_ pseudovirus entry (**E**) and Env-mediated cell-cell fusion (**F**). The pseudovirus entry in TZM-bl cells was determined by a single-cycle infection assay, in which the wild-type (WT) and mutant pseudoviruses were normalized to a fixed amount by p24 antigen. The luciferase activity of wild-type virus was treated as 100%, and the relative infectivity of the mutant virus was calculated. A *t* test was performed to judge the significance of the difference between the WT and mutant and *p* values are shown. Kinetics of HIV-1 Env-mediated cell-cell fusion was determined by a dual split-protein assay (DSP). For both viral entry and fusion, data were derived from the results of three independent experiments and are expressed as means with standard deviations (SD).

In order to define the function of Asn-145 for HIV-1 infectivity, we firstly generated a mutant Env bearing N145A mutation with a plasmid encoding the X4-tropic virus HIV-1_NL4-3_ Env (clade B) as a template. After the site-directed point mutation was verified by DNA sequencing, both the wild-type (WT) and mutant Env-pseudotyped viruses were generated and their infectivity in TZM-bl cells was compared by a single-cycle infection assay. As shown in Figure 2A, the N145A mutant virus exhibited significantly decreased cell entry efficiency relative to the WT virus. Then we measured the kinetics of Env-mediated cell-cell fusion by a dual split-protein (DSP)-based fusion assay. As shown in Figure 2B, the fusogenic ability of the N145A Env also decreased markedly. To verify the results, we further introduced the N145A mutation into two R5-tropic Envs derived from HIV-1_SVPB13_ (clade B) and HIV-1_SVPC13_ (clade C). Consistently, both the mutant Envs displayed significantly reduced activities to mediate pseudovirus entry and cell-cell fusion (Figure 2C–F). Therefore, the conserved residue Asn-145 plays critical roles for HIV-1 Env-mediated cell fusion and infection.

### 3.2. Asn-145 is Critical for the Interactions of the N- and C- Terminal Heptad Repeats (NHR and CHR) Helices

**Figure 3:**
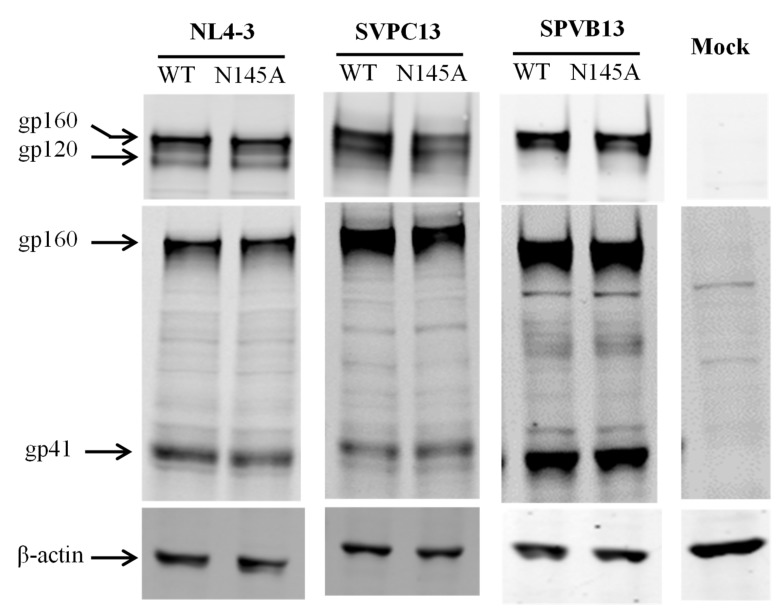
Effects of the N145A mutation on the expression of HIV-1 Env glycoprotein determined by Western blotting. The viral Env glycoproteins in the lysates of transfected cells were detected with a rabbit anti-gp120 polyclonal antibody (upper panel) and the human anti-gp41 monoclonal antibody 10E8 (middle panel). The reaction bands corresponding to gp160, gp120, and gp41 are marked. The experiments were repeated three times, and representative data are shown.

**Figure 4:**
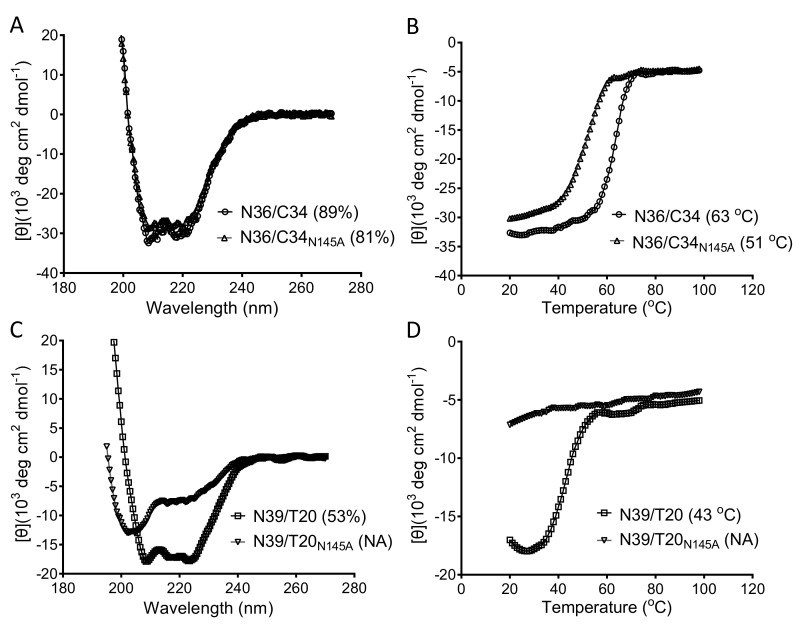
Effects of the N145A mutation on the secondary structure and thermostability of 6-HB. The α-helicity (**A**) and thermostability (**B**) of the native and mutant C34 in complexes with N36 and the α-helicity (**C**) and thermostability (**D**) of the native and mutant T20 in complexes with N39 were respectively determined by circular dichroism (CD) spectroscopy. The final concentration of each peptide was 10 μM in PBS. The experiments were repeated two times, and representative data are shown.

We sought to explore the mechanisms underlying the impaired functionality of Env caused by the N145A mutation. First, we characterized the expression and processing of the Env glycoproteins by Western blotting, in which a rabbit anti-gp120 polyclonal antibody and the human anti-gp41 monoclonal antibody 10E8 were used to probe the cleaved (gp120/gp41) and uncleaved (gp160) Env glycoproteins. As shown in Figure 3, no obvious changes were found in the three Envs bearing the N145A mutation. Next, we characterized whether the N145A mutation affect the 6-HB structure that represents a fusion-active core structure of gp41 and plays essential roles in viral fusion and entry. To this end, we synthesized the NHR-derived peptide N36 and the CHR-derived peptide C34 with or without the N145A substitution, and then analyzed their interactions by circular dichroism (CD) spectroscopy. As shown in Figure 4A and B, both C34 and C34_N145A_ interacted with N36 to display a typical α-helical conformation; however, the N145A substitution resulted in apparently reduced α-helicity and thermostability. Specifically, the N36/C34 complex showed 89% α-helices with a melting temperature (*T*_m_) value of 63 ℃, whereas the N36/C34_N145A_ complex showed 81% α-helices with a *T*_m_ of 51 ℃. Because the recent studies suggested that the interactions between the FPPR of NHR and the TRM of CHR also critically determine the NHR-CHR interactions and HIV-1 entry [14,22,34], we further used the NHR-derived peptide N39, the inhibitor T20, and its N145A mutant as surrogates. As shown in Figure 4C-D, the N39/T20 complex displayed 53% α-helices with a *T*_m_ of 43 ℃, but the α-helical content and *T*_m_ of the N39/T-20_N145A_ complex could not be defined, which suggested that the N145A substitution disrupted the helical interaction between N39 and T20.

**Figure 5:**
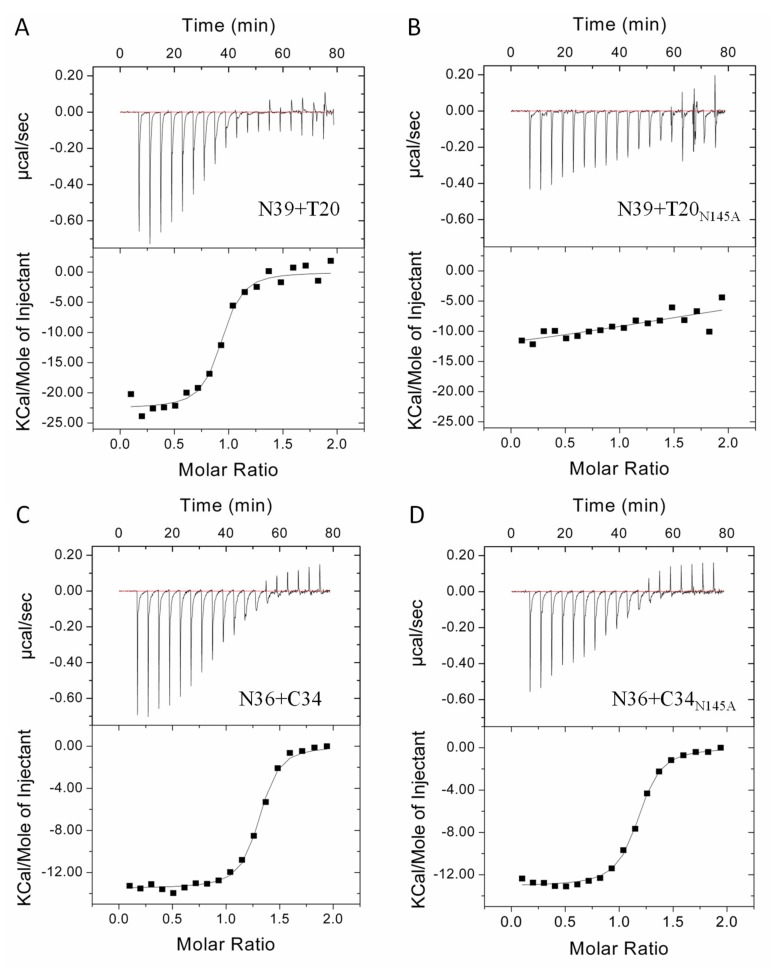
Effects of the N145A mutation on the interactions of the N- and C- terminal heptad repeats (NHR and CHR) helices determined by isothermal titration calorimetry (ITC). Thermodynamic profiles of the molecular interactions between N39 and T20 (**A**), N39 and T20_N145A_ (**B**), N36 and C34 (**C**), and N36 and C34_N145A_ (**D**) were respectively measured. The titration traces are shown at the top, and the binding affinities are shown at the bottom. The experiments were repeated two times, and representative data are shown.

Considering that the CD spectroscopy measured the secondary structure and thermostability of a preformed peptide complex rather than their instantaneous interactions, we next applied isothermal titration calorimetry (ITC) to determine the thermodynamic parameters of the peptide pairs in a real-time molecular interaction, including the binding constant (*K*), reaction stoichiometric (*N*), enthalpy (Δ*H*), and entropy (Δ*S*). As shown in Figure 5 and Table 1, the interactions of all the four peptide pairs were a typical enthalpy-driven reaction, in which large amounts of heat were released. Although the N39/T20 complex had much less α-helicity and thermostability than the N36/C34 complex determined by CD spectroscopy, they exhibited comparable binding affinities with the *K* values of 3.2 ×10^6^ M^-1^ and 3.3 × 10^6^ M^−1^, respectively (Figure 5A,C), which verified that non-helical interactions might dominate the interactions between N39 and T20. Surprisingly, the N39 and T20 interaction could be severely impaired by the N145A substitution, resulting in an unstable reaction profile in which thermodynamic parameters could not be precisely defined (Figure 5B); by contrast, C34_N145A_ largely retained its interaction affinity with N36 with a *K* value of 2.2 × 10^6^ M^−1^. Taken all the biophysical data together, we conclude that the polar Asn-145 residue plays critical roles in the interaction of the NHR and CHR helices of gp41.

### 3.3. Asn-145 Determines the Antiviral Activity of Fusion Inhibitors

**Figure 6:**
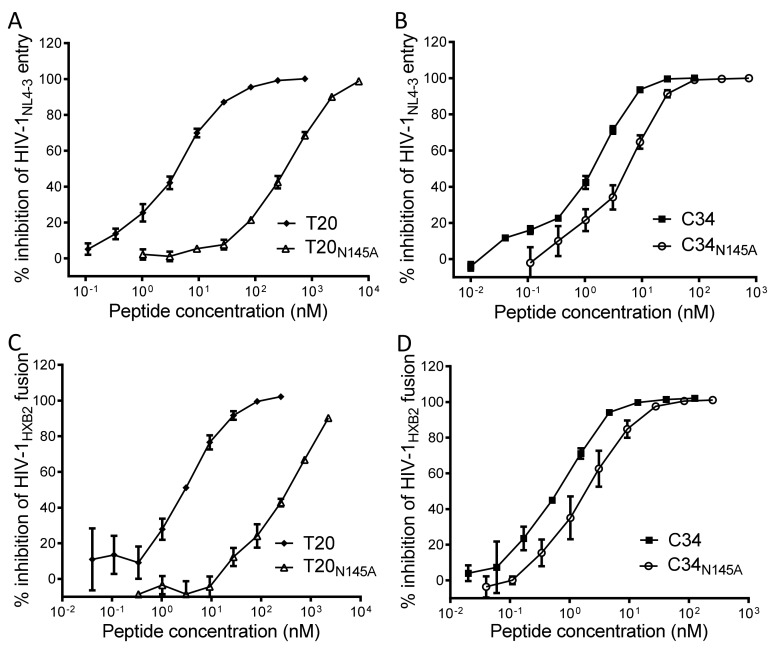
Effects of the N145A mutation on the anti-HIV activity of C-terminal heptad repeats (CHR-derived) fusion inhibitors. The inhibitory activities of T20 and T20_N145A_ (**A**) C34 and C34_N145A_ (**B**) on HIV-1_NL4-3_ pseudovirus were compared by a single-cycle infection assay. The inhibitory activities of T20 and T20_N145A_ (**C**) C34 and C34_N145A_ (**D**) on HIV-1_HXB2_ Env-mediated cell-cell fusion were compared by a reporter gene assay with TZM-bl cells as a target and HL2/3 cells as an effector. The experiments were conducted in triplicate and repeated three times. Percent inhibition of the inhibitors and IC_50_ values were calculated. Data are expressed as means ± SD.

Both T20 and C34 are CHR-derived native peptides with potent anti-HIV activity. Herein, we focused on determining whether the N145A substitution affected the inhibitory activities of T20 and C34. In the single-cycle infection assay, T20 and T20_N145A_ inhibited HIV-1_NL4-3_ pseudovirus entry in TZM-bl cells with IC_50_ values of 4.2 ± 0.5 and 291.5 ± 26 nM, respectively, which indicated a 69.4-fold reduction for the inhibitory activity of T20_N145A_ (Figure 6A); C34 and C34_N145A_ inhibited HIV-1_NL4-3_ pseudovirus with IC_50_ of 1 ± 0.1 and 4.8 ± 0.7 nM, respectively, indicating a 4.8-fold reduction for the inhibitory activity of C34_N145A_ (Figure 6B). In the cell-cell fusion assay, T20 and T20_N145A_ inhibited HIV-1_HXB2_ Env-mediated cell fusion with IC_50_ of 4 ± 0.7 and 304.1 ± 58.6 nM, respectively, which indicated a 76-fold difference (Figure 6C); C34 and C34_N145A_ inhibited HIV-1_HXB2_ with IC_50_ of 0.6 ± 0.1 and 2.2 ± 0.5 nM, respectively, indicating a 4-fold difference (Figure 6D). Therefore, Asn-145 is also an important residue for the antiviral activity of CHR-based fusion inhibitor peptides.

### 3.4. Asn-145 Serves as a Turning-Point for the Anti-HIV Activity of Helical Peptide Inhibitors

**Figure 7:**
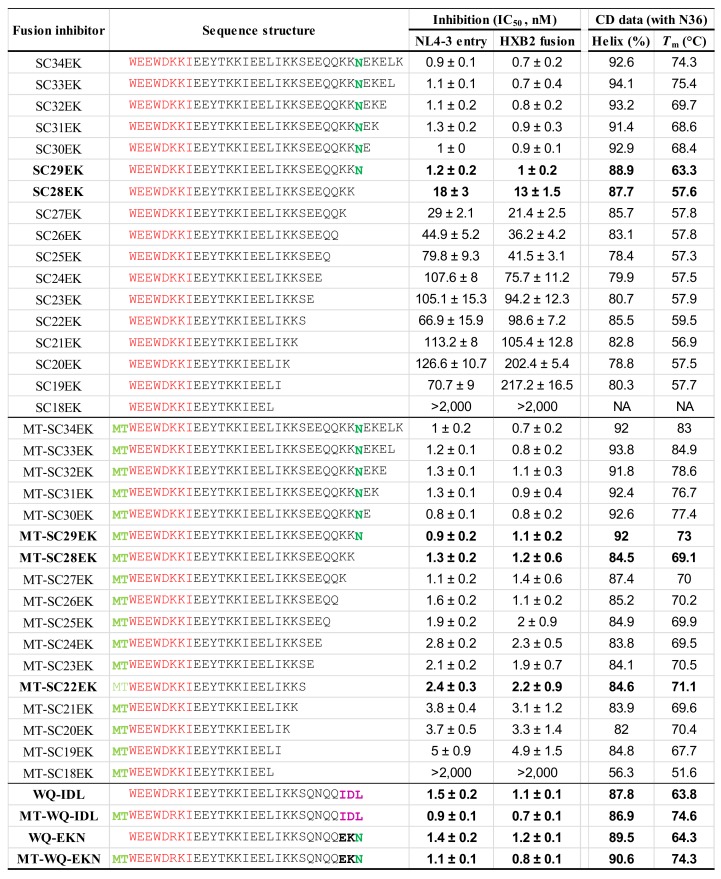
Structural and functional characterization for the functionalities of Asn-145 in helical peptide inhibitors. The inhibitory activities of electrostatically constrained inhibitors on HIV-1_NL4-3(D36G)_ pseudovirus entry and HIV-1_HXB2_ Env-mediated cell–cell fusion were determined, with the experiments performed in triplicate and repeated three times, and data are expressed as means ± SD. The α-helicity and binding thermostability of diverse inhibitors were determined by circular dichroism (CD) spectroscopy, with the final concentration of each peptide at 10 μM in PBS, and the experiments were repeated two times, and representative data are shown. The M-T hook residues, PBD, Asn-145, and IDL anchor are respectively marked in aqua, red, green, and magenta. NA, not applicable.

To verify the importance of Asn-145 for fusion inhibitors, we synthesized a panel of truncated peptides with the electrostatically constrained C34 peptide SC34EK as a template (Figure 7). The antiviral activities and binding stabilities of all the peptides were characterized in parallel for comparison. It was found that deleting one to five amino acids from the C-terminus of SC34EK had no obvious effects on the inhibitory activities of the peptides against HIV-1_NL4-3_ entry and HIV-1_HXB2_ fusion; however, the deletion of Asn-145 from SC29EK resulted in the inhibitors with sharply reduced anti-HIV potencies. Specifically, SC29EK inhibited HIV-1_NL4-3_ and HIV-1_HXB2_ with IC_50_s of 1.2 and 1 nM, respectively, whereas SC28EK had IC_50_s at 18 and 13 nM, respectively. Interestingly, further deleting amino acids one-by-one from SC28EK only resulted in the inhibitors with modestly decreased activities until SC18EK, which could not tolerate the truncation. As determined by CD spectroscopy, the single Asn-145 deletion also caused a greatly decreased binding stability, evidenced by the *T*_m_ values of SC29EK (63.3 ℃) and SC28EK (57.6 ℃). Surprisingly, the nine amino acids preceding Asn-145 seemly had no functions in terms of peptide’s binding stabilities. In contrast, two amino acids downstream the Asn-145 residue, glutamic acid and leucine, could enhance the binding, as shown by the *T*_m_ values of SC30EK (68.4 ℃) and SC33EK (75.4 ℃); However, the increased binding stabilities had no contributions to the antiviral activity of the inhibitors. We also applied ITC to compare the interactions of SC29EK and SC28EK with N36, which verified the critical roles of Asn-145 in the binding affinity of the inhibitors (Table 1). 

### 3.5. Asn-145 Regulates the Antiviral Function of the M-T Hook Structure

We previously demonstrated that the M-T hook structure can greatly enhance the NHR-CHR interaction and the antiviral activity of HIV-1 fusion inhibitors [18,19,20,35]. Promisingly, short-peptides modified with the M-T hook residues but not containing the Asn-145 residue possessed highly potent anti-HIV activities [31,36,37]. To comprehensively characterize the functionality of Asn-145 and define the structure–activity relationship (SAR) of various fusion inhibitor peptides, we further generated and characterized a panel of peptides by adding two M-T hook residues into the N-terminus of all the corresponding SC34EK derivatives. Very surprisingly, all the new peptides, except the shortest MT-SC18EK, exhibited highly potent and comparable anti-HIV activities (Figure 7). By comparison, the addition of the M-T hook structure dramatically increased the inhibitory activities in the absence of Asn-145, as demonstrated by the peptides from MT-SC28EK to MT-SC19EK; however, it had no or minor roles when Asn-145 were presented, as demonstrated by the peptides from MT-SC34EK to MT-SC29EK. In the presence of the M-T hook residues, the C-terminal 15 amino acids of MT-SC34EK only displayed limited roles in the inhibition of HIV-1 infection, confirming the importance of the M-T hook structure for developing a short-peptide inhibitor with a high anti-HIV activity. As shown by the CD data, all the M-T hook-modified peptides had markedly increased *T*_m_ values (~10 ℃) regardless of the presence or absence of the Asn-145 residue, verifying again that the M-T hook structure can greatly enhance the binding stability of the inhibitors.

### 3.6. Substitution of an IDL Anchor for Asn-145 Slightly Improves the Binding But Not the Inhibitory Activities

**Figure 8:**
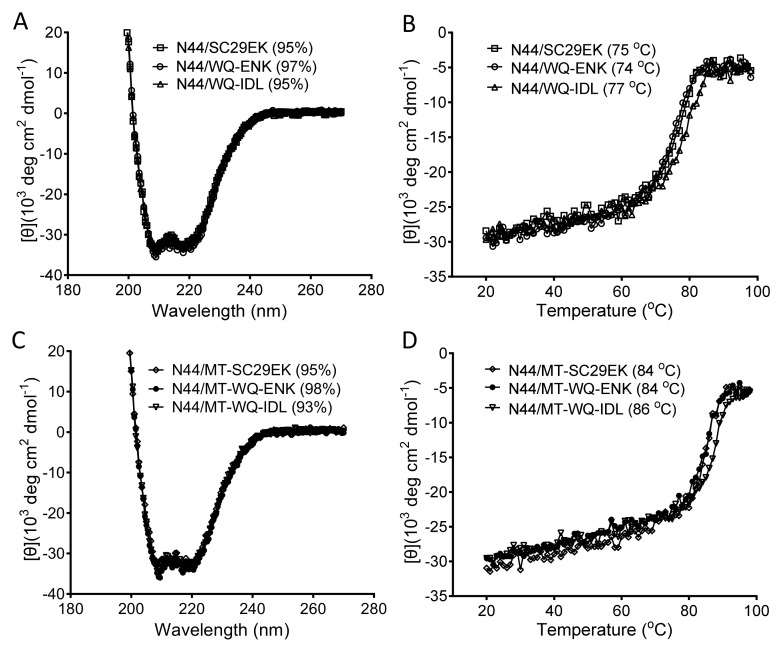
The α-helicity and binding stability of the inhibitors with a C-terminal Asn-145 or IDL anchor. The α-helical contents (**A**) and thermostabilities (**B**) of SC29EK, SC29-ENK, and WQ-IDL in the presence of the NHR-derived target mimic peptide N44 and the α-helical contents (**C**) and thermostabilities (**D**) of the M-T hook-modified inhibitors MT-SC29EK, MT-SC29-ENK, and MT-WQ-IDL in the presence of N44 were respectively determined by circular dichroism (CD) spectroscopy. The final concentration of each peptide was 10 μM in PBS. The experiments were repeated two times, and representative data are shown.

Recently, Su and coworkers identified a shallow pocket in the N-terminal region of the NHR trimer (termed N-pocket) and intentionally designed fusion inhibitors that can fit this site [26,27,28]. By replacing three C-terminal residues of SC29EK- and MT-SC29EK-based peptides with an artificial IDL (Ile-Asp-Leu) anchor, the authors generated two new peptides (designated WQ-IDL and MT-WQ-IDL) with significantly increased anti-HIV activities. Herein, we were interested to directly compare them with the parental peptides containing Asn-145. To achieve a maximum sequence similarity, the peptides WQ-EKN and MT-WQ-EKN were also synthesized. As shown in Figure 7, WQ-IDL displayed very similar inhibitory and binding activities with those of SC29EK and WQ-EKN, whereas MT-WQ-IDL did similarly with those of MT-SC29EK and MT-WQ-EKN. Because N36 missed the N-pocket-forming residues, we thus synthesized the target mimic peptide N44 to measure the binding thermostabilities of the inhibitors. As shown in Figure 8, the peptides with the IDL anchor did have slightly increased *T*_m_ values (2~3 ℃) over the parental ones. Therefore, the results indicate that the C-terminal Asn-145 residue and IDL anchor have comparable functionalities in SC29EK sequence-based inhibitors.

### 3.7. Structural Basis for the Functionality of Asn-145 in SC29EK

**Figure 9:**
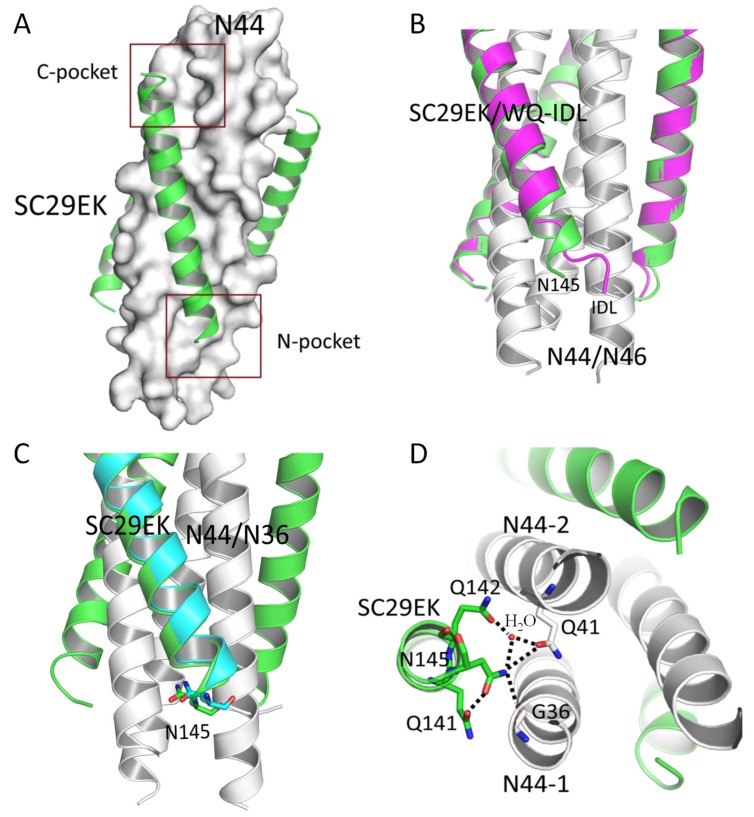
Structural basis of Asn-145-mediated binding network in SC29EK inhibitor. (**A**) The 6-HB structure of the SC29EK/N44 complex. The N44 trimer is shown in surface and colored in gray, with the C-terminal deep pocket (C-pocket) and the N-terminal shallow pocket marked; SC29EK inhibitors are shown in ribbon and colored in green. (**B**) Superimposing of the SC29EK/N44 structure with the WQ-IDL/N46 structure (PDB accession number 5H0N). SC29EK inhibitors are in green with a marked Asn-145 residue, WQ-IDL inhibitors are in magenta with a marked IDL anchor. (**C**) Superimposing of the SC29EK/N44 structure with the SC29EK/N36 structure (PDB accession number 5Z0W). SC29EK in the SC29EK/N44 is marked in green and SC29EK in the SC29EK/N36 is in cyan. (**D**) Stick model of Asn-145-mediated interhelical and intrahelical interactions. Dashed lines indicate the hydrogen bonds.

We were intrigued to clarify the structure–function relationship of Asn-145 in the context of fusion inhibitor peptides. It was reported that the IDL tail adopted a hook-like conformation to fit the N-pocket when the inhibitors were complexed with N43 or N46, but it adopted an extended α-helical conformation when complexed with N36 that lacks the N-pocket-forming residues [26,28]. To investigate the mechanism underlying SC29EK-induced resistance mutations, we previously solved the crystal structure of SC29EK complexed with N36, in which Asn-145 was confined to the extended helix [38]. Based on our observations above, we wondered whether the C-terminal residues of SC29EK interact with the N-pocket similar to that of the IDL anchor. Thus, we determined the crystal structure of SC29EK complexed with N44 that possesses the N-pocket-forming sequence. The space group of the crystal complex belonged to P2_1_2_1_2_1_, and each asymmetric unit contained three pairs of SC29EK/N44 peptides, diffracted x-rays to a resolution of 2.3 Å with an intact electron density map and good refinement values (Table 2). As expected, SC29EK and N44 formed a typical 6-HB structure, in which three N44 formed a NHR trimeric coiled coil with three hydrophobic grooves between adjacent helices and three SC29EK helices were accommodated in these grooves in an antiparallel orientation. Both the C-terminal deep pocket and the newly-defined shallow N-pocket could be readily visualized (Figure 9A). Different from the IDL anchor, the C-terminal residues of SC29EK adopted an extended α-helical conformation, similar to those in the SC29EK/N36 complex (Figure 9B,C). Asn-145 was located at the “*a*” position of the CHR helices, faced the inner NHR helices, and mediated a plenty of intrahelical and interhelical interactions. Specifically, Asn-145 formed an interhelical hydrogen bond with Gly-36 (3.2 Å) located at the “*c*” position of one N44 helix and a hydrogen bond with a water molecule (2.8 Å), which simultaneously donated a hydrogen bond to Gln-41 (2.1 Å) located at the remarkable glutamine-rich polar layer of a neighboring N44 (Figure 9D). Interestingly, the water molecule also donated a hydrogen bond to Gln-142 (2.7 Å) in SC29EK, which further stabilized the interhelical interactions. As illustrated, the NH group of Asn-145 in SC29EK donated a hydrogen bond to the O group of Gln-141, which enhanced the α-helicity of the inhibitor and coordinately contributed to the Asn-145-based interacting network.

## 4. Discussion

We have dedicated the past decade to exploiting the structure and function of HIV-1 gp41 and to developing new viral fusion inhibitors. In highlight, we identified several structural features crucial for the functionality of gp41, including the “QIWNNMT” motif [20], interhelical salt-bridges [16,17], M-T hook structure [18,19], and pocket-2 conformation [21]; a group of new fusion inhibitors were accordingly designed, including sifuvirtide [39], CP32M [35], MT-SC22EK [37], HP23 [31], 2P23 [36], LP-11 [40], LP-19 [41], LP-46 [42], LP-50 and LP-51 [43], LP-52 [44], LP-80 [45], and LP-83 [46]. We also endeavored to select and characterize HIV-1 mutants resistant to the fusion inhibitors, revealing multiple genetic pathways and resistance mechanisms [30,32,38,47,48]. Meanwhile, a large panel of crystal structures were determined for various fusion inhibitors, including CP32 [19], CP32M [49], sifuvirtide [50], MT-C34 [18], MT-SFT [51], SC22EK and MT-SC22EK [37], HP23L and LP-11 [52], SC29EK [38], LP-40 [53], LP-46 [42], and very recently, T20 [22]. These series data have definitely provided important information for understanding the mechanism of gp41-dependent membrane fusion and facilitated the development of novel anti-HIV drugs. In this study, we focused on characterizing the extremely conserved polar residue Asn-145 located at the CHR of gp41 and achieved significant findings. First, we demonstrated by mutational analysis that Asn-145 plays critical roles for gp41 to mediate HIV-1 fusion and entry. Second, it was found that the substitution of Asn-145 does not affect the expression and processing of viral Env glycoproteins but interferes with the interactions between the NHR and CHR helices of gp41, underlying its mechanism of action in viral infectivity. Third, Asn-145 was verified to be important for the antiviral activity of fusion inhibitors and serve as a turning-point for both the inhibitory and binding functions of α-helical peptide-based inhibitors. Fourth, the results suggested that Asn-145 can regulate the functionality of an N-terminal M-T hook structure in fusion inhibitor peptides, and it acts similarly with a C-terminal IDL anchor to confer the inhibitory potency. Fifth, we further determined the crystal structure of the α-helical peptide inhibitor SC29EK complexed with an NHR-derived target mimic peptide, which revealed Asn-145-mediated interhelical and intrahelical interactions in details.

It is well documented that molecular interactions between the NHR and CHR helices of gp41 are essential for HIV-1 fusion and infection. By mutational analysis, a large number of amino acids in the NHR and CHR sequences have been functionally characterized, especially those located at the positions mediating the interhelical interactions as predicted with helical wheel and hairpin models [54,55,56,57,58,59,60,61]. However, Asn-145 has been not specifically focused on by previous studies, even though it is an extremely conserved CHR residue in all the HIV-1, HIV-2, and SIV isolates. In the present study, the mutational analyses with the Envs derived from three classes of HIV-1 isolates demonstrated the importance of Asn-145 for viral cell fusion and entry. It was frequently observed that single amino acid substitutions in gp41 could severely affect the expression and processing pattern of the Env glycoproteins, being a potential mechanism responsible for the damaged or disrupted viral infectivity. Herein, the N145A substitution had no such effects; rather, it might harm the fusogenicity of gp41 through interfering with the packing interactions of the NHR and CHR helices, especially that between the FPPR and TRM sequences, as revealed by CD spectroscopy and ITC experiments (Figure 4 and Figure 5). Previous crystal structures of the gp41 core have identified the detailed molecular contacts within the trimer of hairpin structure (6-HB), which represents a key feature of gp41 and thus it has been used to depict the current HIV-1 fusion model and to guide the design of fusion inhibitors [3,4,9,10,14]. With an intention to visualize the binding conformation of Asn-145 in SC29EK, which is an electrostatically constrained helical peptide inhibitor, we determined the crystal structure of SC29EK in the presence of N44, an NHR-derived target surrogate. Asn-145 does not interact with Val-38 in the NHR helix as predicted by the helical wheel and hairpin models; instead, it contacts directly or indirectly with the Gly-36 and Gln-41 residues. This inspired our curiosity to know the binding properties of Asn-145 in context of a viral 6-HB, we thus analyzed the crystal structures of the N36/C34 complex and a large recombinant gp41 protein containing the FPPR and TRM sequences. Both the structures verified that Asn-145 interacts with the Gly-36 and Gln-41 residues in the same way as Asn-145 does in the SC29EK inhibitor (data not shown). Therefore, the helical packing interactions between the NHR and CHR helices of gp41 are highly complicated and remain to be characterized in more details.

Despite considerable efforts, T20 remains the only membrane fusion inhibitor available for the treatment of viral infection; however, its clinical application has been largely limited owing to its low anti-HIV potency, short in vivo half-life, and susceptibility to drug resistance [62,63,64,65]. Various strategies are applied to develop novel HIV-1 fusion inhibitors with significantly improved pharmaceutical profiles. For examples, introducing mutations that facilitate peptide’s α-helicity and solubility by electrostatic constraints [39,66,67]; applying protease-resistant D-amino acids or β-amino acids [68,69]; conjugating peptides with membrane-binding lipids [40,42,53,70,71]; adding an M-T hook structure to the N-terminus of an inhibitor that enhances its binding with the deep NHR pocket [18,51,72]; and incorporating an IDL anchor in the C-terminus of an inhibitor to fit the shallow N-pocket [26,27,28]. In all the cases, the CHR-derived inhibitors T20 and C34 were used as design templates. In this study, we demonstrated that Asn-145 plays crucial roles for T20 and C34, as its alanine substitution resulted in the peptides with a greatly reduced binding and inhibitory activities. Importantly, Asn-145 was identified as a turning-point for the antiviral potency by gradually truncating the electrostatically constrained peptide SC34EK. Clearly, the deletion of the C-terminal five amino acids from SC34EK had little effects on its inhibitory potency, but its Asn-145 could not be further truncated, verifying SC29EK being the minimum inhibitor with a potent anti-HIV activity among this class of peptides [67,73].

Previous studies demonstrated that the M-T hook structure can greatly enhance the binding and inhibitory activities, thus providing a new strategy for designing HIV-1 fusion inhibitors [18,29,51,72]. By adding two M-T hook residues, several highly potent short-peptides have been developed, which mainly target the deep NHR pocket site [31,36,37]. Herein, we clarified that Asn-145 has a regulatory role on the functionalities of the M-T hook structure. In the presence of Asn-145, the addition of two M-T hook residues markedly increased the binding stability of the inhibitors but contributed little to the inhibitory activity. In sharp contrast, the M-T hook structure was a critical determinant for both the binding and inhibition in the absence of Asn-145 (Figure 7). Therefore, the present data confirmed again that the M-T hook structure is a powerful strategy, especially for the development of short-peptide-based HIV-1 fusion inhibitors. In this study, we also generated data to clarify that Asn-145 has similar functionalities with the IDL anchor for the anti-HIV activity of an inhibitor with or without the M-T hook. While the IDL anchor was reported to penetrate the N-pocket formed by four hydrophobic NHR amino acids (Leu-33, Leu-34, Ile-37, and Val-38) [26,28], our crystallographic studies revealed that Asn-145 is still with the extended α-helical conformation and its side chain makes substantial interhelical and intrahelical interactions to stabilize the binding, thus calling in question for an IDL anchor in this site. Indeed, while the M-T hook can dramatically strengthen the binding stability of inhibitors with the large and deep pocket in the C-terminal site of the NHR trimer, the IDL anchor only slightly improves the binding, which also queries the potential of such a small and shallow N-pocket being an ideal target site, especially for short-peptide fusion inhibitors.

As illustrated by the canonical helical wheel and hairpin models in Figure 1, the “*a*” position residue Asn-145 on the CHR helix should interact with the “*e*” position residue Val-38 on the NHR helix. Indeed, the previous crystal structures of 6-HBs revealed that Asn-145, together with Leu-149 and Glu-146, can form a hydrophobic site, in which the side chain of Val-38 interacts with [3,4,18,50]. However, both the structures of SC29EK in complexes with N44 and N36 found that Asn-145 might not make hydrophobic interactions with the Val-38 residues. It is conceivable that without the downstream Leu-149 and Glu-146 residues the single polar Asn-145 located at the C-terminal extreme of SC29EK inhibitor could not provide a local hydrophobic environ; instead, it largely makes extensive interactions with the target site through a hydrogen-bond network, dominated by a glutamine-rich polar layer. Taking all the results together, our studies also endorse the continuous efforts to exploit the mechanisms of gp41-dependent HIV-1 fusion/entry and of action of fusion inhibitors.

## Figures and Tables

**Table 1:** Thermodynamic parameters of the interactions between the N- and C- terminal heptad repeats (NHR- and CHR-derived) peptides determined by isothermal titration calorimetry (ITC).

Peptide Pair	*N*	*K* (M^−1^)	△*H* (Kcal/mol)	△*S* (cal/mol/deg)
N39/T20	0.9	3.2 × 10^6^	−22.6	−45.9
N39/T20_N145A_	2.6	3.6 × 10^4^	−13.5	−30.7
N36/C34	1.3	3.3 × 10^6^	−15.4	−15.5
N36/C34_N145A_	1.1	2.2 × 10^6^	−13.1	−14.8
N36/SC29EK	1.1	2.6 × 10^6^	−15.8	−23.6
N36/SC28EK	1.3	5.2 × 10^5^	−9.0	−3.66

**Table 2:** Crystallographic data collection and refinement statistics.

Parameter	Value *^a^*
Data Collection	
Beamline	SSRF BL17U
Wavelength (Å)	0.97915
Resolution rage	45.000–2.33 (2.47–2.33)
Space group	*P*2_1_2_1_2_1_
Cell dimesions	
a, b, c (Å)	36.500, 39.860, 171.503
a, b, g (°)	90.00, 90.00, 90.00
Redundancy	3.19
Total no of reflections	66,040
No. of unique reflections	20,721
*R*_merge_*^b^*(%)	8.3 (86.1)
I/SIGMA	10.16 (1.53)
Completeness (%)	98.0 (90.6)
**Refinement**	
Resolution (Å)	38.825–2.330 (2.44–2.33)
No. of reflections	11,172
*R*_work_/*R*_free_*^c^*	0.2330/0.2859
No. of atoms	
Protein	1762
Water	10
B factors (Å^2^)	
Protein	67.28
Water	60.77
RMS deviations	
Bond lengths (Å)	0.003
Bond angles (°)	0.491
Ramachandran plot (%)	
Favored	97.95%
Allowed	1.54%
Disallowed	0.51%

*^a^* Values in parentheses are the statistics in the highest-resolution shell. *^b^ R*_merge_ =∑*_hkl_*∑_j_ |I*_hkl,j_*- I *_hkl_* |/∑ *_hkl_*∑*_j_* I*_hkl,j_*, and I*_hkl_* is the average of symmetry-related observations of a unique reflection. *^c^ R*_work_ = ∑*_hkl_*||*F*_obs_|−|*F*_calc_||/∑*_hkl_*|*F*_obs_|, where *h*, *k*, and *l* are the indices of the reflections and *F*_obs_ and *F*_calc_ are the observed and calculated structure factors deduced from the model, respectively. *R*_free_ is defined as cross-validation *R* factor for 5% of reflections against which the model was not refined.
